# A Thermometrical Table on the Scales of Fahrenheit, Centigrade, and Reaumur; Comprising the Most Remarkable Phenomena Connected with Temperature in Relation to Climatology, Physical Geography, Chemistry and Physiology

**Published:** 1845-04

**Authors:** 


Art. IX.—*A Thermometrical Table on the Scales of Fahrenheit, Centigrade, and Reaumur ; comprising the most remarkable phenomena connected with temperature in relation to Climatology, Physical Geography, Chemistry, and Physiology.* By Alfred S. Taylor, Lecturer on Chemistry, &c. in Guy's Hospital.—*London,* 1845.

If ever there was a book entitled to the character of a *multum in parvo*, this assuredly is the book, if book it may be called which book is none, but merely an engraved TABLE, with a brief printed preface. But, whatever be its appropriate name, it most assuredly contains in a single page a mass of matter, so vast, that it might easily be expanded into a goodly-sized volume,—and matter, too, highly important, very varied, constantly needed, and nowhere else to be found in a collected form. In a word, we can boldly pronounce this little work to be one of those provokingly useful things which must be bought, and which the law of convenience and comfort makes almost as necessary to us in these lazy latter days, as was the food and the drink in times " when wild in woods the noble savage ran." To give some idea of the number and kind of things here recorded, we may state that the observer will see *at a glance* almost every important fact relating to temperature, in climatology, physical geography, chemistry, physiology, &c. The following are the particulars given, under the heads Chemistry, and Physiology :

" Chemistry. 1. The evaporating, boiling, fusion, melting, subliming and congealing points of all solids and liquids in chemistry, from 12° to 374° F., from — 11° to + 190° C. and from — 9 to + 152° R., including the boiling points of the saturated solutions of numerous salts, and the melting points of a large number of alloys. 2. The temperature for fermentation of various kinds, malting, putrefaction, etherification, and other chemical processes. 3. The boiling points of alcohol and acids of various specific gravities, with the respective densities of the vapours. 4. The pressure or elastic force of the vapour of water, alcohol, oil of turpentine, and ether, at various temperatures. 5. The temperatures, with the corresponding pressures required for the liquefaction of the gases. 6. The temperature for the explosion and ignition of fulminating and combustible substances.

Physiology. 1. The maximum degrees of natural and artificial heat, and minimum degrees of cold, borne by man and animals. 2. The temperature of the body in Man, Mammalia, Birds, Reptiles, Fishes, and Insects. 3. The temperature at which hybernation takes place in certain animals. 4. The temperature for the germination of seeds, incubation, the artificial hatching of the ova of birds, fishes and insects. 5. The temperature for the growth of the Sugar cane, Date, Indigo, Cotton-tree, and for the cultivation of the Vine. 6. The temperature for warm, tepid and vapour baths; the vapour baths of Russia and Finland." (pp. 8-10.)

